# Therapeutic effect of dichloroacetate against atherosclerosis via hepatic FGF21 induction mediated by acute AMPK activation

**DOI:** 10.1038/s12276-019-0315-2

**Published:** 2019-09-30

**Authors:** Byong-Keol Min, Chang Joo Oh, Sungmi Park, Ji-Min Lee, Younghoon Go, Bo-Yoon Park, Hyeon-Ji Kang, Dong Wook Kim, Jeong-Eun Kim, Eun Kyung Yoo, Hui Eon Kim, Mi-Jin Kim, Yong Hyun Jeon, Yong-Hoon Kim, Chul-Ho Lee, Jae-Han Jeon, In-Kyu Lee

**Affiliations:** 1 Department of Biomedical Science, Graduate School and BK21 plus KNU Biomedical Convergence Programs, Daegu, South Korea; 2 Research Institute of Aging and Metabolism, Kyungpook National University, Daegu, South Korea; 3 Leading-edge Research Center for Drug Discovery and Development for Diabetes and Metabolic Disease, Kyungpook National University Hospital, Daegu, South Korea; 4 Korean Medicine Application Center, Korea Institute of Oriental Medicine, Daegu, South Korea; 5 Laboratory Animal Center, Daegu-Gyeongbuk Medical Innovation Foundation, Daegu, South Korea; 6 Laboratory Animal Resource Center, Korea Research Institute of Bioscience and Biotechnology, Daejeon, South Korea; 7 Department of Internal Medicine, School of Medicine, Kyungpook National University, Daegu, South Korea

**Keywords:** Atherosclerosis, Dyslipidaemias

## Abstract

Dyslipidemia-induced atherosclerosis, which has a risk of high morbidity and mortality, can be alleviated by metabolic activation associated with mitochondrial function. The effect of dichloroacetate (DCA), a general pyruvate dehydrogenase kinase (PDK) inhibitor, on in vivo energy expenditure in ApoE^−/−^ mice fed a western diet (WD) has not yet been investigated. WD-fed ApoE^−/−^ mice developed atherosclerotic plaques and hyperlipidemia along with obesity, which were significantly ameliorated by DCA administration. Increased oxygen consumption was associated with heat production in the DCA-treated group, with no change in food intake or physical activity compared with those of the control. These processes were correlated with the increased gene expression of *Dio2* and *Ucp-1*, which represents brown adipose tissue (BAT) activation, in both WD-induced atherosclerosis and high-fat-induced obesity models. In addition, we found that DCA stimulated hepatic fibroblast growth factor 21 (*Fgf21*) mRNA expression, which might be important for lowering lipid levels and insulin sensitization via BAT activation, in a dose- and time-dependent manner associated with serum FGF21 levels. Interestingly, *Fgf21* mRNA expression was mediated in an AMP-activated protein kinase (AMPK)-dependent manner within several minutes after DCA treatment independent of peroxisome proliferator-activated receptor alpha (PPARα). Taken together, the results suggest that enhanced glucose oxidation by DCA protects against atherosclerosis by inducing hepatic FGF21 expression and BAT activation, resulting in augmented energy expenditure for heat generation.

## Atherosclerosis: Expending surplus energy on cardiovascular health

A compound that shifts cellular energy consumption into inflation confers effective protection against cardiovascular diseases. Elevated lipid put people at risk of atherosclerosis and coronary artery disease, but new findings from researchers led by In-Kyu Lee at Kyungpook National University in Daegu, South Korea reveal a potential strategy for preventing such accumulation. They have demonstrated that a small molecule called dichloroacetate targeting for mitochondrial respiration boosts metabolic activity and impedes atherosclerosis progression in a experimental animal model fed Western diets. Dichloroacetate might work by stimulating signals that activate brown adipose tissue, a type of fat that burns excess nutrients for energy rather than storing them. These signaling pathways have been linked to other aspects of obesity and cardiovascular disease, suggesting dichloroacetate may be a promising drug candidate.

## Introduction

Excess nutrient-induced dyslipidemia and diabetes are significantly associated with cardiovascular diseases, including atherosclerosis and coronary artery disease^1^. Many clinical and basic studies have demonstrated that primary therapeutics for vascular diseases regulate dyslipidemia, which is characterized by increased triglyceride (TG) and low-density lipoprotein (LDL) cholesterol levels as well as low plasma concentrations of high-density lipoprotein (HDL), which precede cardiovascular diseases related to oxidative stress^2–5^. The role of brown and beige adipocytes as metabolically active tissues in combating obesity-related metabolic diseases including type II diabetes and atherosclerosis through plasma TG/cholesterol clearance and glucose disposal, which is mediated by uncoupling protein 1 (UCP-1), and related with energy expenditure^6,7^ has been highlighted. Classically, brown adipose tissue (BAT) activation mediated by β-adrenergic agonists can alleviate the development of atherosclerosis by reducing the LDL content in the blood^8^. Thus, hyperlipidemia is the main risk factor for atherosclerosis, which might be therapeutically rescued by the clearance of lipoprotein triglyceride-derived fatty acids in activated metabolic tissues such as BAT and beige fat.

Many studies have demonstrated that fibroblast growth factor (FGF) 21 is a therapeutic agent for the treatment of obesity-related diseases, including atherosclerosis, which increases lipoprotein uptake and catabolism^9–11^. It is secreted predominantly by the liver, is upregulated by starvation, the consumption of a ketogenic diet, and amino acid deprivation via peroxisome proliferator- activated receptor alpha (PPARα)- or activating transcription factor 4 (ATF4)-dependent mechanisms, and binds to FGF receptor 1 and β-Klotho. This leads to increases in thermogenic genes, the β-oxidation of lipids and the metabolic rate through PPAR gamma coactivator 1α (PGC1α) in BAT^12,13^. FGF21 plays an important role in energy-expending processes by inducing PGC1α-mediated thermogenic genes in the mitochondria in most adipose tissues in an autocrine/paracrine manner^14,15^. However, elevated serum FGF21 is positively associated with carotid atherosclerosis in humans, especially in women, suggesting that it is a biomarker of or a therapeutic target for atherosclerosis disease, although this is controversial^16^. Recent studies have proposed that mitokines related to muscle-specific mitochondrial dysfunction, including FGF21 and growth differentiation factor 15, can systematically protect against obesity and insulin resistance through a mitohormetic signal involving ATF4, which regulates integrated stress response^17–19^. An engineered FGF21 variant, as a promising therapeutic candidate, ameliorates methionine choline-deficient diet-induced nonalcoholic steatohepatitis progression, as evidenced by increased lipid-utilizing mitochondrial respiration in hepatocytes^20^. There is evidence indicating that BAT stimulation is required for an increase in energy expenditure, as confirmed by the increased uptake of ^18^F-fludeoxyglucose (^18^F-FDG) specifically by intercapsular BAT in mice fasted overnight and hence in a noninsulin-stimulated state, which is indicative of heightened energy demand^7,21^. The collective findings indicate that FGF21 has a mitohormetic effect in response to various metabolic disorders that are positively associated with mitochondrial dysfunction in adaptive thermogenesis.

Mitochondrial dysfunction leads to excessive lactate production, reactive oxygen species (ROS) production, and a deficient ATP supply due to the reduced oxidative phosphorylation of glucose. Therefore, the mitochondrial pyruvate dehydrogenase complex has been implicated as a central metabolic node that regulates the conversion of pyruvate into acetyl-CoA rather than lactate through pyruvate dehydrogenase kinase (PDK) and allosteric inhibition by acetyl-CoA, NADH, and ATP^22^. Dichloroacetate (DCA) is a rapid-acting small molecule that targets mitochondrial PDK and is effective in preventing lactic acidosis with no beneficial effect on hemodynamics or survival^23^. However, DCA also significantly improves post-ischemic heart function and attenuates balloon injury-induced vascular restenosis, vitamin D_3_-induced vascular calcification, and acute kidney injury^24–27^. Khan et al. reported that DCA, in addition to improving glucose oxidation, might increase LDL uptake by upregulating LDLR in the liver^28^. To our knowledge, no study has assessed the effect of DCA in an in vivo atherosclerosis model; furthermore, the functional relationship between DCA and hepatic FGF21 expression has not been explored mechanistically. In this study, we report that DCA systematically ameliorates atherosclerosis via FGF21-mediated enhanced energy expenditure in an AMPK-dependent manner, which was confirmed by increased glucose uptake associated with enhanced energy expenditure.

## Materials and methods

### Experimental animals

The procedures used in this study were approved by the Animal Care and Use Committee of Daegu-Gyeongbuk Medical Foundation and Kyungpook National University School of Medicine and were conducted according to the National Institutes of Health Guide for the Care and Use of Laboratory Animals. Male ApoE^−/−^ (B6.129P2, backcrossed with C57BL/6J) and PPARα^−/−^ (008154) mice were obtained from The Jackson Laboratory (Bar Harbor, USA), and male C57BL/6 J mice were obtained from DooYeol Biotech (Seoul, South Korea).

The ApoE^−/−^ mice were fed a western diet (WD, D12079B; Research Diets Inc., New Brunswick, USA) to establish the atherosclerosis model. DCA (100 or 150 mg/kg) was administered by oral gavage once per day after 4 weeks of WD. For PET/CT analysis, C57BL/6J mice were fed with a high-fat diet (HFD, D12492; Research Diets Inc., New Brunswick, USA) from 8 weeks of age for 4 or 8 weeks. To explore the chronic effect of DCA on BAT function, 8-week-old C57BL/6 J mice were fed a HFD for 4 weeks and then given DCA in drinking water at a concentration of 1 g/L for another 10 weeks. To assess the effect of DCA mediated by the pharmacological inhibition of PDK, male 8-week-old normal chow-fed PDK2/4 double KO (DKO) mice, in which mitochondrial respiration is increased by PDC activity^29^, were killed to establish cultures of primary brown adipose cells.

### Mouse primary hepatocyte culture

Primary hepatocytes were isolated by the collagenase digestion of liver tissue obtained from 8- to 12-week-old male mice. Cells (2 × 10^6^) were plated on collagen-coated plates with William’s medium E (W4128; Sigma-Aldrich, St. Louis, USA) containing 10% FBS, 100 U/mL penicillin, and 100 μg/mL streptomycin. The cells were cultured for 4 h in 60-mm collagen-coated dishes and allowed to attach. The medium was then replaced with 199 medium (M4530; Sigma-Aldrich, St. Louis, USA) supplemented with 23 mM HEPES, 10 nM dexamethasone, 10% FBS, 100 U/mL penicillin, and 100 μg/mL streptomycin.

### Quantitative measurement of atherosclerotic lesions

Mice were euthanized with pentobarbital sodium (50 mg/kg, i.p.), the right atria were removed, and the hearts and aortas were perfused with saline through the left ventricle. Aortas were dissected from the proximal ascending aorta to the bifurcation of the iliac artery, and the adventitial fat was removed. For en face analysis, aortas were split longitudinally, pinned onto flat black silicone plates, and fixed in 4% paraformaldehyde in PBS overnight. Fixed aortas were stained with oil red O for 30 min, washed briefly with distilled water, and digitally photographed. The aortic plaque area was quantified by ImageJ software (NIH).

### Serum analysis

The serum levels of TG, total cholesterol, HDL, LDL, aspartate transaminase (AST), alanine transaminase (ALT), and creatinine were analyzed with an automated serum analyzer (7020; Hitachi, Japan) following the manufacturer’s instructions. Serum VLDL and FGF21 levels were measured by using mouse ELISA kits from CUSABIO (CSB-E17089m) and R&D Systems (MF2100) according to the manufacturer’s instructions.

### Positron emission tomography/computed tomography (PET/CT) imaging

Two hours before PET/CT analysis, DCA (100 mg/kg) was administered by i.p. injection. In vivo PET/CT and CT imaging was performed as described previously^30,31^. All mice were anesthetized with 1–2% isoflurane gas during imaging. PET results were analyzed with anatomical CT images by using 3D image visualization and VIVID software (Gamma Medica-Ideas). Briefly, PET scanning was performed for 10 min by using LabPET8 (TriFoil imaging) followed by CT scanning. To quantify the ^18^F-FDG uptake (% ID/cc) in BAT, the volume of BAT from each CT image was manually determined and analyzed by using PMOD 3.5 software (PMOD Technologies). For CT imaging analysis, the distribution of visceral fat tissue was manually determined, and 3D-rendered CT imaging was prepared by using VIVID software.

### Metabolic phenoCages

To measure metabolic parameters, which included VO_2_, VCO_2_, and physical activity, the mice were housed individually in indirect calorimetric cages (TSE PhenoMaster) as previously reported^31^. Each mouse was monitored for 48 h in the fed state, and all the results were analyzed for 24 h on average. The respiratory exchange ratio and energy expenditure were calculated by using VO_2_ and VCO_2_.

## Results

### DCA ameliorates atherosclerotic plaque formation and dyslipidemia in an atherosclerotic mouse model

**Fig. 1:**
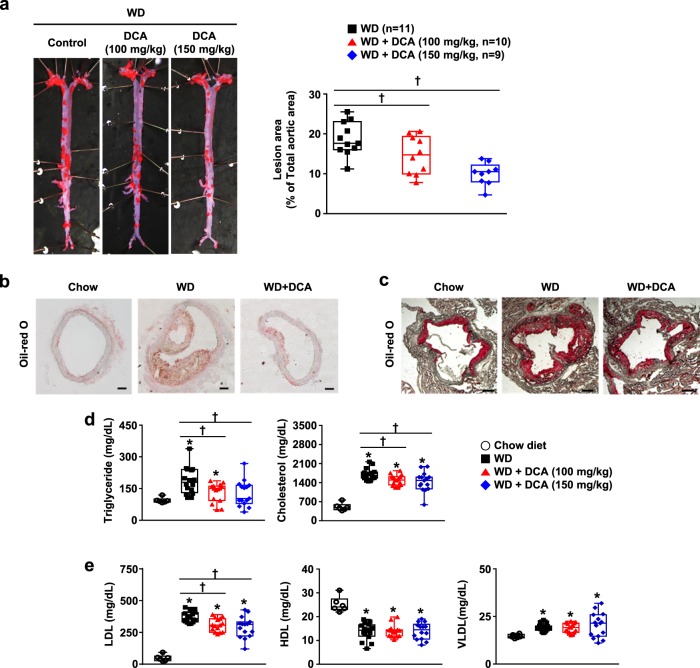
DCA protects against atherosclerotic development in a WD-fed ApoE^−/−^ mouse model. **a** Representative en face images of whole aortas stained with oil red O and the quantification of the aortic plaque area (*n* = 9–11 per group). **b**, **c** Cross sections of aortic sinuses and heart valves from 30-week-old mice stained with oil red O (**b**, scale bar = 100 µm, **c**, scale bar = 200 µm). **d** Serum triglyceride and cholesterol levels and **e** serum HDL, LDL, and VLDL levels (chow-fed group, *n* = 6; WD-fed group, *n* = 15–16). The values are expressed as the mean ± SEM. Statistical analysis was performed by Student’s *t-*test. *p < 0.05 vs. the chow-fed group, ^†^*p* < 0.05 vs. the WD-fed group

To examine the beneficial effect of DCA on atherosclerosis in vivo, we established an atherosclerosis model by using ApoE^−/−^ mice fed with a WD for 16–22 weeks. The entire descending aorta was isolated and stained with oil red O to evaluate the atherosclerotic lesion area. The aortic plaque area was decreased in DCA-treated mice in a dose-dependent manner (21.8 ± 8.0% and 46.8 ± 5.0% at 100 and 150 mg/kg, respectively) compared with that in the WD control group (Fig. 1a). Likewise, the lipid portions of lesions from both aortic cross sections and aortic roots were drastically attenuated in the DCA-treated groups (Fig. 1b, c).

Consistent with previous studies^7,8^, lipid accumulation in atherosclerotic regions was closely associated with dyslipidemia in the blood. According to serum analysis, TG levels were twofold higher in the WD group than in the chow-fed group, and this increase was blocked by 150 mg/kg DCA. Likewise, the total cholesterol level was slightly decreased by 15.7 ± 5.4% in the DCA (150 mg/kg) group (Fig. 1d). Moreover, the significant increase in the LDL level by WD was reduced by DCA treatment in a dose-dependent manner, while the reduced HDL and increased VLDL levels were not altered by DCA treatment among DCA-treated and WD control mice (Fig. 1e). These results demonstrate that DCA prevents atherosclerotic indications resulting from decreased serum TG, LDL, and cholesterol levels in a diet-induced atherosclerotic model.

### DCA reduces the inflamed fat mass by inducing thermogenesis

**Fig. 2:**
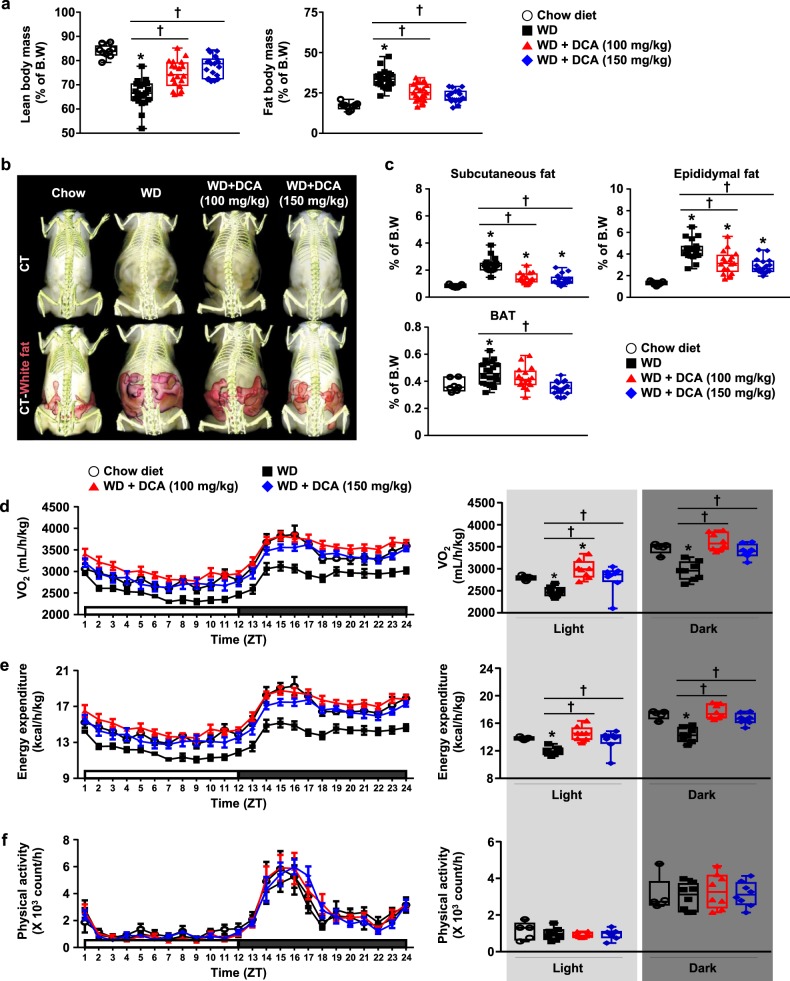
DCA leads to reduced adiposity and higher energy expenditure in WD-fed ApoE^−/−^ mice. **a** Body compositions of mice ascertained by Minispec (chow-fed group, *n* = 9; WD-fed group, *n* = 19–21). **b** Body composition analysis of mice by micro-CT. Visceral fat tissue is highlighted in pink. **c** Fat tissue weight (chow-fed group, *n* = 7; WD-fed group, *n* = 17–20). **d**–**f** Metabolic parameters of the mice. The levels of VO_2_ (**d**), energy expenditure (**e**), and physical activity (**f**) (chow-fed group, *n* = 5; WD-fed group, *n* = 8). The values are expressed as the mean ± SEM. Statistical analysis was performed by Student’s *t*-test. *p < 0.05 vs. the chow-fed group, ^†^*p* < 0.05 vs. the WD-fed group

To investigate how DCA ameliorates atherosclerotic plaques and dyslipidemia, we examined body fat mass associated with the development and progression of WD-induced atherosclerosis. Fat composition in the DCA-treated groups was significantly attenuated, which was consistent with the reduction in body weight compared with that in the WD group. Likewise, the relative lean mass composition was significantly restored in DCA-treated mice (Fig. 2a and online supplementary material, Fig. S1a). Full-body scans by PET–CT imaging indicated that DCA decreased abdominal fat in a dose-dependent manner (Fig. 2b). The weight of subcutaneous fat (40.7 ± 3.9 and 45.0 ± 3.9%) and epididymal fat (26.0 ± 5.7 and 34.2 ± 3.8%) was significantly reduced in the 100 and 150 mg/kg DCA-treated groups, respectively, compared with the WD group (Fig. 2c). Likewise, the augmentation of BAT in the mice fed with a WD, like histological morphology by hematoxylin and eosin staining, was completely restored by 150 mg/kg DCA treatment (online supplementary material, Fig. S1b).

Next, we assessed the metabolic, behavioral, and physiological changes in individual mice fed with a WD for 20 weeks by using metabolic PhenoCages. Food intake was not changed by DCA treatment (online supplementary material, Fig. S2a). Interestingly, the decreased oxygen consumption rate and energy expenditure induced by a WD were significantly restored by 100 mg/kg DCA, which was consistent with a chow diet (Fig. 2d, e and online supplementary material, Fig. S2b). Similarly, the respiratory exchange ratios (RERs) were significantly increased by DCA treatment (online supplementary material, Fig. S2c), suggesting increased glucose oxidation in the DCA-treated groups. However, physical activity during the day and night was not changed among the four groups (Fig. 2f). Collectively, these results support the view that DCA reduces the increased body fat mass by regulating the energy expenditure of ApoE^−/−^ mice fed with a WD.

### Beneficial effect of DCA is mediated by acute and chronically activated BAT

**Fig. 3:**
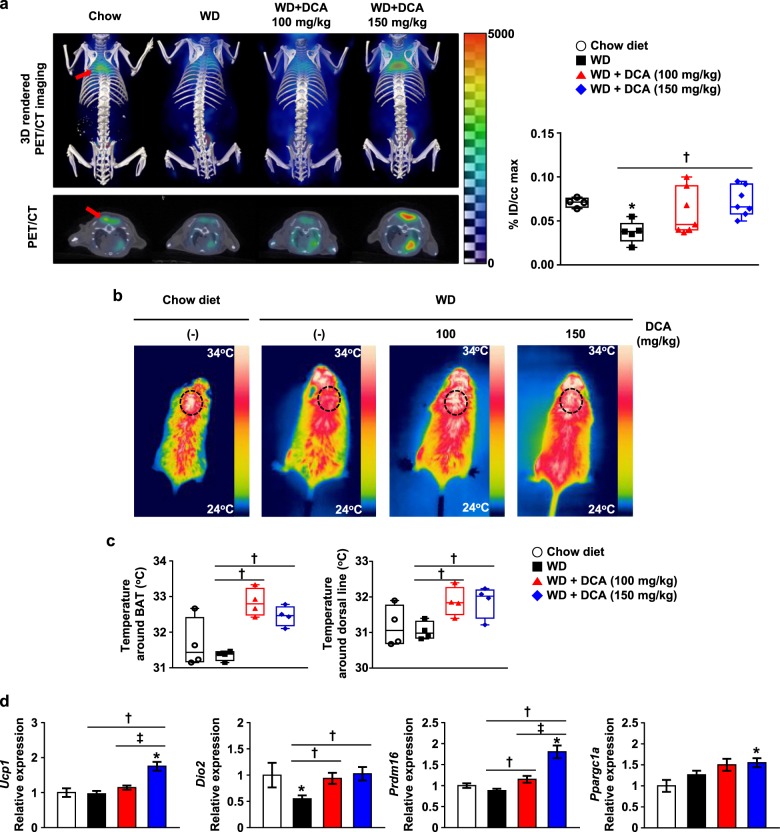
DCA enhances energy consumption for heat generation in atherosclerosis models. **a** 3D-rendered imaging and transverse imaging of ^18^F-FDG uptake in BAT (left). The quantification of ^18^F-FDG uptake in BAT (right). The BAT portion is indicated by the red arrow (chow-fed group, *n* = 4; WD-fed group, *n* = 5–7). **b** Infrared images of the mice. **c** Temperature around BAT and the dorsal midline of the mice, as determined by infrared camera images. The BAT portion is indicated by the dotted circle (*n* = 4 per group). **d** BAT marker gene mRNA expression level in BAT (chow-fed group, *n* = 5; WD-fed group, *n* = 15–16). The values are expressed as the mean ± SEM. Statistical analysis was performed by Student’s *t*-test. *p < 0.05 vs. the chow-fed group, ^†^*p* < 0.05 vs. the WD-fed group, ^‡^*p* < 0.05 vs. the DCA (100 mg/kg)-treated group

BAT thermogenesis plays a crucial role in energy balance regulation by dissipating excess calories^21,32^. DCA treatment dramatically reduced BAT weight in addition to reducing other adipose depots, and this was associated with increased heat generation and energy expenditure. Therefore, we determined whether the main effect of DCA is mediated by BAT activation by using the ^18^F-FDG–PET/CT technique associated with intensifying glucose utilization for thermogenesis in the presence of DCA administration. Glucose uptake by BAT was decreased in mice fed with a WD compared with mice fed with a chow diet, but was markedly increased in DCA-treated mice in a dose-dependent manner (Fig. 3a). Furthermore, thermogenic capacity, which was represented by the temperature around BAT and the dorsal line, was significantly increased in DCA-treated mice based on infrared image analysis (Fig. 3b, c). In accordance with the visualization of activated BAT, the mRNA expression of BAT marker genes such as *Ucp-1*, *Dio2*, and *Prdm16* and lipolysis enzymes including *Hsl* and *Mgl* was also upregulated in BAT from DCA-treated mice compared with mice fed with a WD, while the mRNA expression of *Ppargc1a* appeared to be increased, but there was no significant difference between WD- and DCA-treated mice (Fig. 3d, and online supplementary material, Fig. S3).

**Fig. 4:**
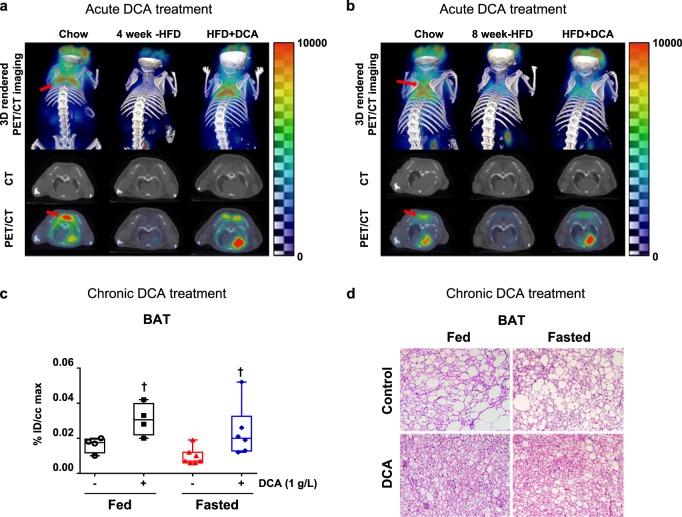
Both the acute and chronic administration of DCA rescues HFD-induced BAT inactivation. **a**, **b** 3D imaging and transverse imaging of ^18^F-FDG uptake in BAT after 4 (**a**) or 8 weeks (**b**) of HFD exposure. The BAT portion is indicated by the red arrow. **c** ^18^F-FDG uptake quantification in BAT after 14 weeks of HFD exposure, which included DCA (1 g/L) treatment via drinking water for the final 10 weeks (fed group, *n* = 4; fasted group, *n* = 6–7). **d** Representative images of hematoxylin and eosin staining of BAT after 14 weeks of HFD exposure, which included DCA (1 g/L) treatment via drinking water for the final 10 weeks (scale bar = 100 µm). The values are expressed as the mean ± SEM. Statistical analysis was performed by Student’s *t* -test. ^†^*p* < 0.05 vs. the WD-fed group

Furthermore, we assessed the acute effect of DCA on BAT activation in mice fed with a 60% high-fat diet (HFD). Compared with 4 or 8 weeks of HFD exposure alone, acute DCA treatment induced BAT activation (Fig. 4a, b). Likewise, after a total of 14 weeks of HFD exposure, including cotreatment with DCA for 10 weeks of HFD exposure, the DCA-treated group exhibited BAT activation under both the fed and fasted conditions (Fig. 4c). Similar to the results of the WD-fed ApoE^−/−^ mouse model, DCA improved the changes in the morphology of BAT induced by HFD (Fig. 4d). The data indicate that the beneficial effect of DCA is mediated by the activation of BAT in mice fed with both a WD and a HFD, and that this might be associated with the restoration of energy expenditure reduced by overnutrition.

### DCA induces hepatic FGF21 expression and FGF21 signaling in BAT

**Fig. 5:**
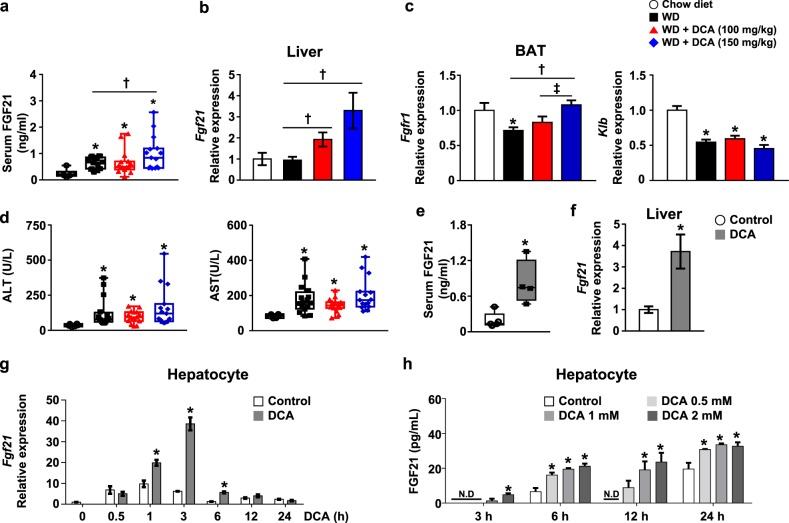
DCA upregulates hepatic FGF21 expression, resulting in BAT activation. **a**, **b** Serum FGF21 concentration (**a**) and *Fgf21* mRNA levels in the liver (**b**) of ApoE^−/−^ mice (chow-fed group, *n* = 5–6; WD-fed group, *n* = 15–16). **c** *Fgfr* and *Klb* mRNA levels in BAT (chow-fed group, *n* = 5; WD-fed group, *n* = 15–16). **d** Serum ALT and AST concentrations (chow-fed group, *n* = 6; WD-fed group, *n* = 15–16). **e**, **f** FGF21 in the serum (**e**) and *Fgf21* mRNA expression in the liver (**f**) after oral DCA (100 mg/kg) administration for 1 week (**e**, *n* = 8 per group; **f**, *n* = 4–5 per group). **g** *Fgf21* mRNA expression level in mouse primary hepatocytes after DCA (1 mM) treatment for the indicated time. **h** Concentration of FGF21 secreted from mouse primary hepatocytes after DCA treatment for the indicated time at the indicated dose. The values are expressed as the mean ± SEM (**a**–**f**) or ± SD **g**–**h**. Statistical analysis was performed by Student’s *t-*test. *p < 0.05 vs. the chow-fed group or the DCA untreated group, ^†^*p* < 0.05 vs. the WD-fed group, ^‡^*p* < 0.05 vs. the DCA (100 mg/kg)-treated group

To identify the molecular mechanism by which DCA ameliorates atherosclerosis via BAT activation in vivo, we hypothesized that DCA induces FGF21, which is predominately expressed in and secreted from the liver, and has a strong effect on UCP-1 expression, resulting in fatty acid oxidation in BAT, as demonstrated previously^33^. First, we assessed serum FGF21 levels, which were slightly increased by a WD and further increased by DCA treatment in a dose-dependent manner (Fig. 5a). Hepatic *Fgf21* mRNA expression was not different between chow-fed and WD-fed mice. However, the hepatic *Fgf21* mRNA level in DCA-treated mice was significantly increased compared with that in WD-fed mice (Fig. 5b). Generally, FGF21 receptors are as important as FGF21 expression for FGF21 signaling, and the mRNA expression levels of the FGF21 receptor (*Fgfr1*) and the coreceptor β-Klotho (*Klb*) were also investigated^34^. We found that the decrease in *Fgfr1* mRNA expression induced by a WD was markedly restored in the BAT of DCA-treated mice, while the decrease in *Klb* mRNA was not significantly different between WD-fed mice and mice treated with either of the two doses of DCA (Fig. 5c). In addition, we evaluated the effect of DCA administration on hepatocellular injury, and we observed that the increase in ALT and AST levels in the serum of WD-fed mice was not improved by DCA treatment (Fig. 5d). Furthermore, we found that DCA directly increased mitochondrial respiration mediated by UCP-1 and PGC1α and FGF21 expression, which was correlated with an increase in the oxygen consumption rate induced by DCA treatment (Fig. S4c), in differentiated mouse primary brown adipocytes at the indicated time points (online supplementary material, Fig. S4a, b).

Next, we evaluated the effect of DCA on hepatic FGF21 expression under chow diet conditions for 1 week in vivo. DCA significantly increased the FGF21 level in the serum, which was consistent with the increases in hepatic *Fgf21* mRNA expression observed in DCA-treated mice (Fig. 5e, f). Likewise, DCA obviously increased *Fgf21* mRNA expression to the greatest extent at 3 h, but these effects faded with time (Fig. 5g). Secreted FGF21 levels were significantly increased by DCA in a dose- and time-dependent manner (Fig. 5h). Interestingly, secreted FGF21 was detected 3 h after the administration of 1 and 2 mM DCA, and significant *Fgf21* mRNA induction was detected within 1 h, suggesting de novo biosynthesis of FGF21 in primary hepatocytes. However, this induction of FGF21 mRNA expression was still comparable to that in PDK2/4 DKO hepatocytes (online supplementary material, Fig. S5a). Hepatic FGF21 expression was significantly increased in the fasted state compared with the fed state despite PDHE1α activity being highly suppressed (online supplementary material, Fig. S5b, c), suggesting that mitochondrial PDHE1α activity is limited to fine-tuning the effect of DCA on FGF21 expression under both fasted and fed conditions.

### DCA increases FGF21 expression irrespective of PPARα

**Fig. 6:**
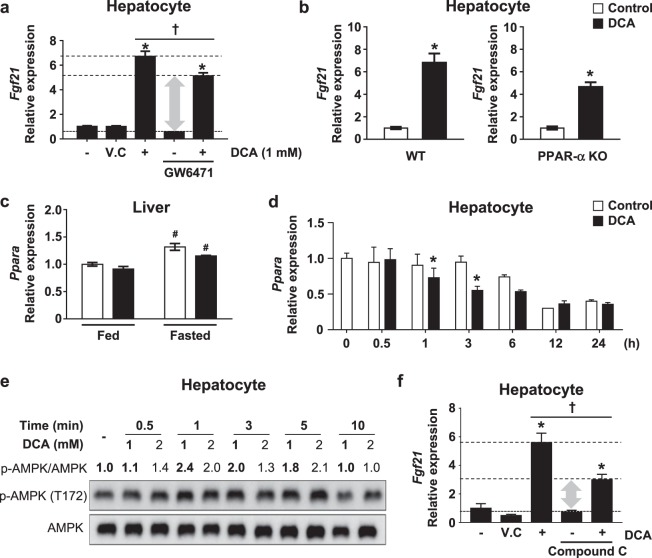
DCA induces hepatic Fgf21 mRNA expression partially through PPARα and AMPK. **a** The effect of 15 min of pretreatment with the PPARα antagonist GW6471 (10 µM) on *Fgf21* mRNA expression. **b** *Fgf21* mRNA expression level in WT or PPARα^−/−^ mouse primary hepatocytes after DCA (1 mM) treatment for 3 h. **c** *Pparα* mRNA expression in the liver of fed and fasted animals after DCA (100 mg/kg) administration for 1 week. **d** *Pparα* mRNA expression in mouse primary hepatocytes after DCA (1 mM) treatment for the indicated time (*n* = 3). **e** Phosphorylated AMPK expression in primary hepatocytes after DCA treatment as the indicated dose for the indicated time. Two different whole-blot images are shown in Supplementary Fig. 4. **f** The effect of 30 min of pretreatment with the AMPK inhibitor compound C (10 µM) prior to DCA (1 mM) treatment for 3 h on *Fgf21* mRNA expression. The values are expressed as the mean ± SD (**a**–**c**, **e** and **f**) or ± SEM (**c**). Statistical analysis was determined by Student’s *t* test. *p < 0.05 vs. the control group, ^†^*p* < 0.05 vs. the DCA-treated group. The vehicle control was a 0.1% DMSO solution

The hepatic gene expression of FGF21 is regulated positively by PPARα^35^. Thus, we assessed the direct effect of PPARα on *Fgf21* mRNA expression by using the administration of 10 µM GW6471, a general PPARα antagonist. DCA-induced *Fgf21* mRNA expression was changed by 23.6 ± 7.0% (Fig. 6a). Although basal *Fgf21* mRNA expression was decreased in PPARα^−/−^ mice compared with wild-type mice^35^, DCA treatment still increased *Fgf21* mRNA expression in the absence of PPARα (Fig. 6b). In addition, *Pparα* mRNA expression was not different in the livers of fed and fasted mice after DCA treatment, and *PPARα* mRNA expression was slightly decreased by DCA treatment at 1 and 3 h in primary hepatocytes (Fig. 6c, d), suggesting that DCA induces *Fgf21* expression irrespective of the presence of PPARα in the liver.

FGF21 ameliorates several metabolic diseases, such as alcoholic fatty liver disease, diabetic cardiomyopathy, and vascular complications associated with AMPK activation^33^. To uncover the pathway involved in DCA-induced FGF21 expression, we determined whether DCA activates AMPK to induce *Fgf21* mRNA expression. AMPK was evidently increased soon after DCA treatment in a time- and dose-dependent manner (Fig. 6e and online supplementary material, Fig. S4). Compared with DCA treatment alone, the administration of compound C, a well-known AMPK inhibitor, markedly attenuated DCA-induced *Fgf21* mRNA expression within 3 h by 47.6 ± 7.8% (Fig. 6f).

## Discussion

Accelerated atherosclerosis is a major cause of morbidity and death in insulin-resistant states such as obesity and metabolic syndrome, but the underlying mechanisms are poorly understood. To define the mechanisms of atherosclerosis and the potential of DCA as a target to prevent lesion development, we developed a model of foam cell-rich atherosclerosis in WD-fed ApoE^−/−^ mice^36^. DCA ameliorated the atherosclerotic plaques through FGF21-mediated energy expenditure, which was correlated with the activation of BAT. These beneficial effects of DCA on diet-induced atherosclerosis were confirmed by histological examinations of the cardiovascular system, indirect gas calorimetry, and ^18^F-FDG uptake by using PET/CT analysis in vivo. Initially, we revealed that DCA-treated ApoE^−/−^ mice fed with a WD displayed a dramatic decrease in body weight and improved dyslipidemia; this effect was similar to the anti-hyperglycemic effect of metformin, which reduces the VLDL–TG level that results from enhanced VLDL–TG uptake and intracellular TG lipolysis followed by mitochondrial fatty acid oxidation in BAT in a manner that is independent of hepatic VLDL–TG production^37^. There are other mechanisms by which DCA affects atherosclerosis; DCA increases LDL intake by inducing LDL receptor expression, as confirmed by the role of the H3 acetylation of the lysine 27 residue of its promoter in hepatic clearance in addition to the bone marrow and spleen^28^. DCA is also effective in preventing pulmonary arterial hypertension and the post-ischemic dysfunction of the hypertrophic heart, while its effect, which is associated with essential cellular metabolism, is lessened upon delayed intervention^24,38^. The hyperpolarization of the mitochondrial membrane potential, which is resistant to apoptosis, is a target of DCA for the promotion of excessive smooth muscle cell proliferation without re-endothelialization^25^, demonstrating that appropriate mitochondrial function might be regulated by metabolic flexibility. Likewise, DCA might protect contractile smooth muscle cells from a synthetic phenotype during the development of atherosclerosis. Thus, our data suggest that DCA-elicited energy expenditure alleviates diet-induced vessel damage in the development of atherosclerosis.

Like metformin, glucagon/glucagon-like peptide 1 analogs, PPARγ activators, sirtuin 1 activator, and lipoic acid increase FGF21 expression and have beneficial metabolic effects via different mechanisms^33^. The benefits of mild mitochondrial stress in tissues are mediated by the effects of hepatic FGF21 expression on enhanced mitochondrial biogenesis in BAT, consistent with our finding that DCA increases Fgf21 mRNA expression in the liver and BAT in an autocrine/paracrine manner to increase thermogenesis^14,39^. Likewise, the beneficial actions of FGF21 on atherosclerosis were identified by inducing adiponectin production from adipose tissues and decreasing the hepatic expression of the transcription factor sterol regulatory element binding proteinλ, resulting in reduced cholesterol synthesis^10^. Furthermore, FGFR1/β-Klotho activation mediates the enhancement of energy expenditure, insulin sensitization, and the induction of high-molecular-weight adiponectin for the treatment of obesity-related metabolic disorders^40^. Subjects with type 2 diabetes mellitus treated with an engineered FGF21 variant (LY2405319) exhibit clinical improvements, consistent with the attenuation of MCD diet-induced nonalcoholic steatohepatitis in ob/ob mice^20,41^. We demonstrated upregulated glucose uptake by BAT upon both acute and chronic administration of DCA. However, to identify the specific mechanism of BAT activation for mitochondrial β-oxidation, it is necessary to evaluate different tracers, namely, ^18^F-FDG, ^18^F-FTHA, and ^11^C-acetate, for glucose uptake, fatty acid uptake, and oxidative activity, respectively, during DCA administration. However, abnormally increased FGF21 expression in the liver in an insulin-resistant state is normalized by decreased binding of retinoic acid receptor gamma to the promoter of retinoid fenretinide and is associated with improved glucose homeostasis and increased FGF21 expression induced by age-related muscle atrophy, resulting in liver steatosis, proinflammatory responses, and even senescence, suggesting an unfavorable effect of FGF21^42,43^. It remains controversial whether catecholamines such as norepinephrine can be produced by activated macrophages and regulated by β3 adrenergic receptor in sympathetic nerve termini in BAT^44^. Taken together, the data suggest that BAT activation might be a tool for the alleviation of hyperlipidemia by DCA in association with FGF21 induction.

**Fig. 7:**
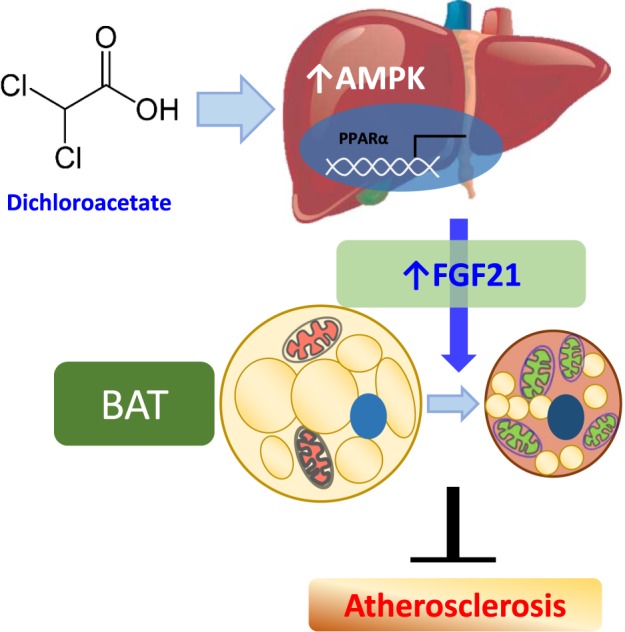
Beneficial effect of DCA on atherosclerosis mediated by hepatic FGF21 associated with both AMPK and PPARα activation

Because DCA reduces fat mass and serum cholesterol levels and enhances thermogenesis correlated with hepatic Fgf21 levels in a dose-dependent manner, FGF21 may act as a mediator of the beneficial effect of DCA on metabolic stress, which is dependent on both AMPK and PPARα signaling in the liver. Many studies of upregulated energy expenditure have implicated the activated AMPK pathway as a key regulator of cellular energy homeostasis required for glucose transport in myocytes as well as mitochondrial UCP-1 upregulation in BAT^45^. The effect of metformin on the suppression of gluconeogenesis is dependent on AMPK, which functions as a conserved cellular energy sensor in an adaptive response to diverse energy stress conditions^46,47^. The mouse Fgf21 promoter includes ATF4-responsive elements and PPAR-responsive elements. An increase in hepatic FGF21 mediated by ATF4 leads to lipolytic gene activation in adipose tissues^48^. FGF21 is significantly nutritionally associated with a protein-restricted diet but not carbohydrate metabolism^33^. In addition, the anti-inflammatory effect of FGF21 in collagen-induced arthritis occurs via anti-oxidative pathways regulated by NRF2 activation in macrophages^49^. Likewise, the increased expression of angiopoietin-like-6 due to mitochondrial oxidative phosphorylation inhibition enhances PPARα-mediated FGF21 expression, which promotes β-oxidation in adipose tissue^50^. Taken together, these results demonstrate that DCA increases Fgf21 mRNA expression, which is mediated mainly by AMPK activation rather than PPARα, as summarized in Fig. 7.

One of the mechanisms of DCA is associated with improved mitochondrial biogenesis and oxidative phosphorylation, which can be explained by mitochondrial quality control. Mitochondrial quality control was recently identified as a beneficial target of the inhibition of dynamin-related protein 1 (Drp1)-mediated mitochondrial fission associated with mitochondrial dysfunction, which is mediated by metformin-induced AMPK-dependent Drp1-mediated mitochondrial fission in endothelial cells^51^. Reduced mitochondrial respiration related to mtDNA copy number, respiratory complex abundance, and oxygen consumption rate has been correlated with causative signs in vascular smooth muscle cells and macrophages in atherosclerosis^52^. In summary, these results suggest that DCA may be therapeutic for patients with atherosclerosis via increasing BAT activation through AMPK-induced FGF21.

## Supplementary information

Supplemental text

Supplemental figures
