# Supplementary material for: Therapeutic effect of dichloroacetate against atherosclerosis via hepatic FGF21 induction mediated by acute AMPK activation
Source: Exp Mol Med. 2019 Sep 30;51(10):114. doi: 10.1038/s12276-019-0315-2 (PMC6802614; doi:10.1038/s12276-019-0315-2)
Supplement: Supplementary file 1 — Supplemental text [file 12276_2019_315_MOESM1_ESM.docx]

**Online-only Data Supplement**

**Therapeutic effect of dichloroacetate against atherosclerosis via hepatic FGF21 induction mediated by acute AMPK activation**

Byong-Keol Min^1*^, Chang Joo Oh^2*^, Sungmi Park^3*^, Ji-Min Lee^1^, Younghoon Go^4^, Bo-Yoon Park^1^, Hyeon-Ji Kang^2^, Dong Wook Kim^3^, Jeong-Eun Kim^3^, Eun Kyung Yoo^3^, Hui Eon Kim^3^, Mi-Jin Kim^2^, Yong Hyun Jeon^5^, Yong-Hoon Kim^6^, Chul-Ho Lee^6^, Jae-Han Jeon^3,7^ and In-yu Lee^1,2,3,7^

^1^Department of Biomedical Science, Graduate School & BK21 plus KNU Biomedical Convergence Programs; ^2^Research Institute of Aging and Metabolism, Kyungpook National University, Daegu, South Korea; ^3^Leading-edge Research Center for Drug Discovery and Development for Diabetes and Metabolic Disease, Kyungpook National University Hospital, Daegu, South Korea; ^4^Korean Medicine Application Center, Korea Institute of Oriental Medicine, Daegu, South Korea; ^5^Laboratory Animal Center, Daegu-Gyeongbuk Medical Innovation Foundation, Daegu, South Korea; ^6^Laboratory Animal Resource Center, Korea Research Institute of Bioscience and Biotechnology, Daejeon, South Korea; ^7^Department of Internal Medicine, School of Medicine, Kyungpook National University, Daegu, South Korea

* These authors contributed equally to this work

**Corresponding author:**

In-Kyu Lee, M.D., Ph.D.

Division of Endocrinology and Metabolism,

Kyungpook National University School of Medicine,

130 Dongdeok-ro, Jung-gu, Daegu, 41944, South Korea

Tel: +82-53-420-5564; Fax: +82-53-426-2046; E-mail: [leei@knu.ac.kr](mailto:leei@knu.ac.kr)

**1. Detailed Methods**

**1.1 Western blot analysis**

Tissue and cells were lysed using a lysis buffer [20 mM Tris (pH 7.4), 10 mM Na_4_P_2_OH, 100 mM NaF, 2 mM Na_3_VO_4_, 5 mM EDTA (pH 8.0), 0.1 mM phenylmethylsulfonyl fluoride, and 1 % NP-40] containing protease inhibitors (aprotinin 7 μg/mL and leupeptin 7 μg/mL) and phosphatase inhibitor cocktail (P0040, Sigma-Aldrich). Protein concentration was measured by BCA protein assay reagent (Thermo Fisher Scientific). Cell lysates were separated by 10 % sodium dodecyl sulfate-polyacrylamide gel electrophoresis and the resolved proteins were transferred to polyvinylidene difluoride membranes (Merck Millipore). The transferred proteins on the membrane were immunoblotted with the following primary antibodies: anti-phospho AMPK (1:1000 in 5 % BSA in TBST), anti-AMPK (1:1000 in 5 % BSA in TBST), and anti-HSP90 (1:1000 in 5 % BSA in TBST).

**1.2 Quantitative real-time polymerase chain reaction (****qRT-PCR)**

Total RNA was extracted from mouse tissue or cells by QIAzol lysis reagent (Qiagen) following the manufacturer’s instructions. Total RNA (2 μg) was used for cDNA synthesis using the cDNA synthesis kit (Thermo Fisher Scientific). qRT-PCR was performed with SYBR Green (Thermo Fisher Scientific) using the ViiA 7 real-time PCR system (Applied Biosystems). Glyceraldehyde 3-phosphate dehydrogenase (GAPDH, for the liver tissue and primary hepatocyte) or 36B4 (for BAT) was used as an endogenous control.

**1.3 Positron Emission Tomography / Computed Tomography (PET/CT) Imaging**

The PET imaging system had the following specifications: ring diameter, 162 mm; field of view (FOV), 60 mm; crystals, 3072; spatial resolution, 1.35 mm full-width half-maximum (FWHM) FOV and noise-equivalent counts, 37 kcps at 245 MBq (250–650 keV). CT scanning was done with an X-ray detector (fly acquisition; 512 projections; binning setting, 2×2; frame number, 1; X-ray tube voltage, 75 kVp; focal spot size, 50 µm; magnification factor, 1.5; matrix size, 512) immediately after PET imaging. PET images were reconstructed by three-dimensional-OSEM iterative image reconstruction and the CT images were reconstructed using filtered back-projections.

**1.4 Thermographic imaging**

The surface body temperature image was collected using an infrared thermographic camera (T620; FLIR, Australia). The temperature of area around BAT and dorsal line were quantified from thermographic images using specific software (FLIR Tools).

**1.5 Quantitative real-time PCR primers**

| **Gene name (mouse)** | **Sequence** |
| --- | --- |
| Ucp1 - Forward | GGTCGTGAAGGTCAGAATGCA |
| Ucp1 - Reverse | GCATTGTAGGTCCCCGTGTAGC50 |
| Dio2 - Forward | TGCACGTCTCCAATCCTGAAT |
| Dio2 - Reverse | GCCCCATCAGCGGTCTT |
| Prdm16 - Forward | AACCAGGCATCCACTCGAAT |
| Prdm16 - Reverse | TGGACACGGTGGGTTGCT |
| Ppargc1a - Forward | TGCGGGATGATGGAGACA |
| Ppargc1a - Reverse | GCGAAAGCGTCACAGGTGTA |
| Fgf21 - Forward | GTACCTCTACACAGATGACGACCAA |
| Fgf21 - Reverse | CGCCTACCACTGTTCCATCC |
| Fgfr1 - Forward | GCCCCTCACCCACAATCC |
| Fgfr1 - Reverse | CGGCAAAGGAGGAGGAGAA |
| Klb - Forward | TGTGGTGAGCGAAGGACTGA |
| Klb - Reverse | GGAGTGGGTTGGGTGGTACA |
| Ppara - Forward | GAACAAAGACGGGATGCTGA |
| Ppara - Reverse | ACAGAACGGCTTCCTCAGGT |
| 36B4 - Forward | ACCTCCTTCTTCCAGGCTTT |
| 36B4 - Reverse | CTCCAGTCTTTATCAGCTGC |
| Gapdh - Forward | GAAGGGTGGAGCCAAAAG |
| Gapdh - Reverse | GCTGACAATCTTGAGTGAGTTG |

**1.6 Antibody**

| **Antibody name** | **Company** | **Catalog No.** |
| --- | --- | --- |
| Phospho-AMPKα (Thr172) | Cell signaling | 2535 |
| AMPKα | Cell signaling | 2532 |

**Supplementary Figure Legends**

**Figure S1. DCA on body weight and tissue histology in WD fed ApoE-/- model.**

(**a** and **b**) Body weight (Chow diet fed group; n=9, WD fed group; n=21–22) (**a**) and Representative images of hematoxylin-eosin staining in metabolic organs (scale bar; 100 μm) (**b**). Values were expressed as mean ± SEM. Statistical analysis was performed by Student’s t test. *p < 0.05 vs. chow diet fed group, †p < 0.05 vs. WD fed DCA only group.

**Figure S2. Metabolic parameters from WD fed ApoE-/- model ± DCA treatment.**

(**a**) Daily food intake in ApoE^-/-^ (each n=17). (**b** and **c**) Metabolic parameters of the mice. The levels of VCO_2_ (**b**) and RER (**c**) (Chow-diet fed group; n=5, WD fed group; n=8). Values were expressed as mean ± SEM. Statistical analysis was performed by Student’s t test. *p < 0.05 vs. chow diet fed group, †p < 0.05 vs. WD fed DCA only group.

**Figure S3. mRNA expressions of lipolysis enzymes in BAT from WD fed ApoE-/- model ± DCA treatment.**

(**a**) Lipolysis related genes including Atgl, Hsl and Mgl mRNA expressions in BAT from the mice. Values were expressed as mean ± SEM. Statistical analysis was performed by Student’s t test. *p < 0.05 vs. chow diet fed group, †p < 0.05 vs. WD fed DCA only group and ‡p < 0.05 vs. DCA (100 mg/kg) treated group.

**Figure S4. DCA may directly activate BAT and induce FGF21 in an autocrine manner.**

(**a** and **b**) The mRNA level of BAT marker genes (**a**) and FGF21 and its corresponding receptor genes (**b**) were measured after DCA (1 mM) treatment for indicated time in differentiated mouse primary brown adipocytes. n=3-4 per each group. (**c**) Oxygen consumption rate (OCR) was measured after DCA treatment for 3 h as indicated dose in differentiated mouse primary brown adipocytes. n=5 per each group. Values were expressed as mean ± SD. Statistical analysis was performed by Student’s t test. *p < 0.05 vs. DCA untreated group.

**Figure S5. DCA-induced FGF21 expression is not dependent on PDK.**

(**a**) Fgf21 mRNA expression level in mouse primary hepatocytes from WT or PDK2/4 DKO after DCA (1 mM) treatment for 3 h. n=2-3. Values were expressed as mean ± SD. Statistical analysis was performed by Student’s t test. *p < 0.05 vs. DCA untreated group. (**b** and **c**) Liver tissue and serum was collected from the mice in fed state or 16 h fasting state. PDHE1a protein phosphorylation level in the liver (**b**) and serum FGF21 concentration (**c**) were measured. n=3-4 per each group. Values were expressed as mean ± SEM. Statistical analysis was performed by Student’s t test. *p < 0.05 vs. DCA untreated group.

**Figure S6. Whole blot images with the size markers in Figure 6E.**

(**a**) Phospho-AMPK (T172). (**b**) AMPK.
