# Supplementary material for: Therapeutic effect of dichloroacetate against atherosclerosis via hepatic FGF21 induction mediated by acute AMPK activation
Source: Exp Mol Med. 2019 Sep 30;51(10):114. doi: 10.1038/s12276-019-0315-2 (PMC6802614; doi:10.1038/s12276-019-0315-2)
Supplement: Supplementary file 2 [file 12276_2019_315_MOESM2_ESM.docx]

## Slide 1

Supplementary  Figure 1

40

Body weight (g)

Time (weeks)

0

35

30

25

20

2

4

6

8

10

12

14

16

*

*

*

†

*

†

†

†

†

†

†

†

†

†

†

†

a

b

Chow

BAT

WD

WD + DCA
100 mg/kg

WD + DCA
150 mg/kg

Subcutaneous
fat

Epididymal
fat

Liver

○ Chow diet
■ WD
▲ WD + DCA (100 mg/kg)
◆ WD + DCA (150 mg/kg)

## Slide 2

Supplementary Figure 2

a

c

○ Chow diet                            ■ WD
▲ WD + DCA (100 mg/kg)       ◆ WD + DCA (150 mg/kg)

0

1

2

3

4

5

Food consumption
(g/day/mouse)

*

*

*

RER

0.7

0.8

1.0

1

2

3

4

5

6

7

8

9

10

11

12

13

14

15

16

17

18

19

20

21

22

23

24

Time (ZT)

b

0.9

RER

0.7

1.0

Light

Dark

0.8

0.9

*

*

†

†

*

*

*

*

VCO2 (ml/h/kg)

1500

2000

2500

3000

3500

4000

1

2

3

4

5

6

7

8

9

10

11

12

13

14

15

16

17

18

19

20

21

22

23

24

Time (ZT)

VCO2 (ml/h/kg)

1500

2000

2500

3000

3500

4000

Light

Dark

*

†

†

*

†

†

*

○ Chow diet
■ WD
▲ WD + DCA (100 mg/kg)
◆ WD + DCA (150 mg/kg)

## Slide 3

Supplementary Figure 3

a

Relative expression
(Atgl)

0

0.5

1.0

1.5

Relative expression
(Hsl)

0

0.5

1.0

1.5

Relative expression
(Mgl)

0

1

2

3

4

5

○ Chow diet
■ WD
▲ WD + DCA (100 mg/kg)      
◆ WD + DCA (150 mg/kg)

‡

†

*

*

*

*

†

†

*

*

†

## Slide 4

Supplementary Figure 4 

Relative expression
(Ucp-1)

0

1

2

3

4

Relative expression
(Prdm16)

0

0.5

1

1.5

Relative expression
(Dio2)

0

0.5

1

1.5

Relative expression
(Pgc1a)

0

0.5

1

1.5

2

a

0

1

3

6

12

24

DCA (h)

0

1

3

6

12

24

DCA (h)

0

1

3

6

12

24

DCA (h)

0

1

3

6

12

24

DCA (h)

*

*

*

*

*

*

*

Relative expression
(Fgf21)

0

1

2

3

4

5

Relative expression
(Fgfr1)

0

0.5

1

1.5

Relative expression
(Klb)

0

0.5

1

1.5

2

b

0

1

3

6

12

24

DCA (h)

0

1

3

6

12

24

DCA (h)

0

1

3

6

12

24

DCA (h)

*

*

*

*

*

*

*

*

● Control
■ DCA 0.5 mM
◆ DCA 1 mM
▲ DCA 2 mM

0

20

40

60

80

Time (minutes)

0

800

OCR (pmol/min)

Rot/AA

FCCP

Oligo

0

250

500

750

OCR (pmol/min)

c

Basal OCR

Maximal OCR

■ Control
■ DCA 0.5 mM
■ DCA 1 mM
■ DCA 2 mM

*

*

*

*

600

400

200

## Slide 5

Supplementary Figure 5

0

2

4

6

Relative expression
(Fgf21)

*

*

■ WT-Control
■ WT-DCA
■ DKO-Control
■ DKO-DCA

a

b

p-PDHE1α
(S300)

PDHE1α

HSP90

Fasted

-

-

+

+

-

-

+

+

DCA (100 mg/kg)

Fed

c

FGF21 (pg/ml)

0

150

300

500

2500

4500

Fasted

Fed

*

*

■ Control
■ DCA

## Slide 6

Supplementary Figure 6

a

b
